# Deciphering the Link Between Mental Health and Green Space in Shenzhen, China: The Mediating Impact of Residents' Satisfaction

**DOI:** 10.3389/fpubh.2021.561809

**Published:** 2021-02-10

**Authors:** Yuhan Qiao, Zini Chen, Yuqing Chen, Tianxiang Zheng

**Affiliations:** Department of Electronic Commerce, Shenzhen Tourism College, Jinan University, Guangzhou, China

**Keywords:** green space, mental health, satisfaction with green-space environment, multilevel regression model, Shenzhen

## Abstract

Rapid urbanization and increasing urban density in China threaten residents' mental health. As a vital component of built environments, green space plays a key role in individuals' psychological well-being; however, the mediating effect of residents' satisfaction with the green space environment on the relationship between urban greening and residents' mental health in Chinese contexts has yet to be thoroughly explored. To fill this knowledge gap, this paper attempts to reveal the internal logic and mechanism underlying the linkages between green space, residents' mental health, and their satisfaction with green space in Shenzhen, China. Specifically, this paper explores the mediating role of residents' satisfaction with a green space environment using questionnaire survey data, “Quick Bird-2” high-resolution remote sensing image data, and a multilevel regression model. Our empirical findings indicate that the relative range of neighboring green spaces can directly improve residents' mental health. More importantly, the relationship between the relative scope of green space and residents' mental health is mediated by residents' satisfaction with the green space environment rather than its direct health effects. Given the influence of green space on residents' satisfaction with the environment, green space indirectly affects mental health. These findings should provide the government useful guidance for considering the spatial distribution and quantity of green space. Our results should also help residents improve their actual experiences and subjective satisfaction with the green space environment.

## Introduction

Rapid urbanization has enlarged China's urban population dramatically; meanwhile, the quality of citizens' living environment has declined (1). Environmental protection is an urgent issue and a fundamental requirement for sustainable development. The construction of ecological civilization has been incorporated into China's national master plan, elevating ecological construction to the national strategic level. As people's living standards have improved, the importance of green space for mental health, especially for stress relief, has attracted the attention of the Chinese public (2, 3). As an aspect of urban construction, greening is responsible for a city's ecological functioning and has been shown to exert significant positive effects on Chinese residents' mental health (4) and well-being (1).

Urban green space, which is typically defined as vegetated open areas such as parks, gardens, playgrounds, and forests (5), has been considered increasingly essential to residents' mental health (6, 7). Over the past decade, research on the relationship between green space and mental health has shifted from qualitative to quantitative designs (8) due to dramatic breakthroughs in quantifying non-physical health benefits (9). Many studies have focused on the pathways through which green space affects mental health. Potential pathways are associated with two primary domains, namely restoring capacities and building capacities (7). For example, green space has been found to relieve residents' stress (10–14), depression (12, 14, 15) and psychological fatigue (10, 11), foster social cohesion (4, 12, 14, 16–18), improve residents' satisfaction (4), build mindfulness and resilience to stress (19), and promote residents' sense of security and adaptability (20). Prolonged exposure to a high-quality natural environment may inspire people to ponder their priorities and goals as well; doing so can help individuals identify new directions in life, which benefits mental health (21). In a recent study, Liu et al. found that stress and neighborhood social cohesion altogether had a complete mediation effect on the association between green exposure and mental health in China (12). Overall, associations between green space and various psychological and emotional benefits have been reported in studies with diverse samples (22–25).

Despite encouraging findings, the mental health benefits of green space for specific population groups or neighborhood settings appear inconsistent. Some researchers have reported correlations that defied their expectations. For example, no relationship has been observed between local green space availability and people's mental health: the presence of more green space was found to be associated with better mental health for men at any age but only for older women (26). No correlation manifested in the Netherlands between neighborhood green space and mental health among housewives or the elderly (27). Other studies have even uncovered adverse effects of green space on residents' mental health: a summary from the World Health Organization (2016) suggested that greater green space may be tied to potential or actual criminal activity (8). The possibility of crime can evoke fear, particularly among vulnerable groups such as women, children, and the elderly (9, 28). In addition, urban greening may raise property rents and taxes in neighboring areas, which could lead to the displacement of individuals with a lower socioeconomic status and evoke hidden risks of mental illness (29).

To date, studies have focused on the impact of residents' satisfaction with green space on their mental health. For example, past work showed that individuals' mental health improved when they were satisfied with green space (30, 31). Liu et al. identified a mediating effect of satisfaction when they investigated the pathways of possible mediators between green space and residents' mental health (4). These studies highlight the need to consider subjective measures of green space quality that may contribute to people's health status. Studies have likewise indicated that green space significantly contributes to neighborhood satisfaction and mental well-being (32). By contrast, Ruijsbroek et al. discovered a weak correlation between individuals' satisfaction with neighborhood green space and mental health (33). Another cross-sectional analysis revealed that green space was related to satisfaction and mental health; however, the specific association was not clear (34). This ambiguity may be due to the lack of a uniform definition of green space and its quality when elucidating green space satisfaction (35). The preceding review indicates that scant studies have explored the correlation between satisfaction derived from the quality of green space and individuals' health outcomes.

The topic of green space and mental health lends itself to case studies. Accordingly, relevant work has involved American, British, and other European cities (e.g., in Denmark); Australia; and the Chinese cities of Beijing and Guangzhou (4, 6, 9, 36). Many other parts of the world (e.g., most of Asia, Africa, less affluent European countries, and South America) have not been the subjects of such research. In light of the diverse ethnic and socioeconomic attributes of such populations, which were identified as confounds in Ruijsbroek et al.'s study (6), findings from focal areas may not necessarily apply elsewhere. These discrepancies cast doubt upon whether residents' emotions will benefit mental health similarly in developing countries compared with Western contexts. For example, most green space is owned and controlled by local governments in China. Given the unique associations between the government, green space, and residents, more specific and process-oriented research is needed to enrich our understanding of the complexities underlying associations between residents' satisfaction with green space and mental health (36).

In summary, evidence pertaining to the relationship between green space and residents' satisfaction and mental health remains thin (36–39). Satisfaction partially reflects the extent to which neighborhood green space enhances residents' mental well-being (4, 14). Even so, the mediating effect of residents' satisfaction with the green space environment on the relationship between urban greening and residents' mental health in Chinese contexts has yet to be thoroughly explored. The present study therefore delineates the relationship between residents' mental health and urban green space in Shenzhen, China, by examining the mediating effect of residents' satisfaction as recommended in prior studies (4, 9). The remainder of this paper is organized as follows: our data collection procedures and methodology are summarized in section Data and Methods; experimental results are described in section Results to reveal the mediating role of residents' satisfaction; and section Conclusion and Discussion presents our discussion and conclusion.

## Data and Methods

### Data

Shenzhen, China was selected as the research area in this study. Shenzhen was a frontier city of China's reform and opening up and is an ideal setting in which to consider the mediating role of residents' satisfaction between green space and mental health. Following more than 30 years of rapid development, Shenzhen has grown into a modern city that stands at the forefront of large and medium-sized cities in China. According to Xinhuanet (40), in 2018, Shenzhen's per capita park and green area covered 16.0 m^2^; its comprehensive air quality index was 3.15; and its environmental noise sound effect level was 57.5, ranking first in China's Green City Index. In August 2019, Shenzhen became the pilot demonstration zone for socialism with Chinese characteristics, continuing to promote green space construction. As of December 2019 (41), the green coverage rate in Shenzhen's built-up area was 43.40%, and more than 2,400 km of greenways spanned the city. However, to our best knowledge, the connection between Shenzhen residents' satisfaction with green space and their mental health remains uncertain and calls for deeper exploration.

**Figure 1:**
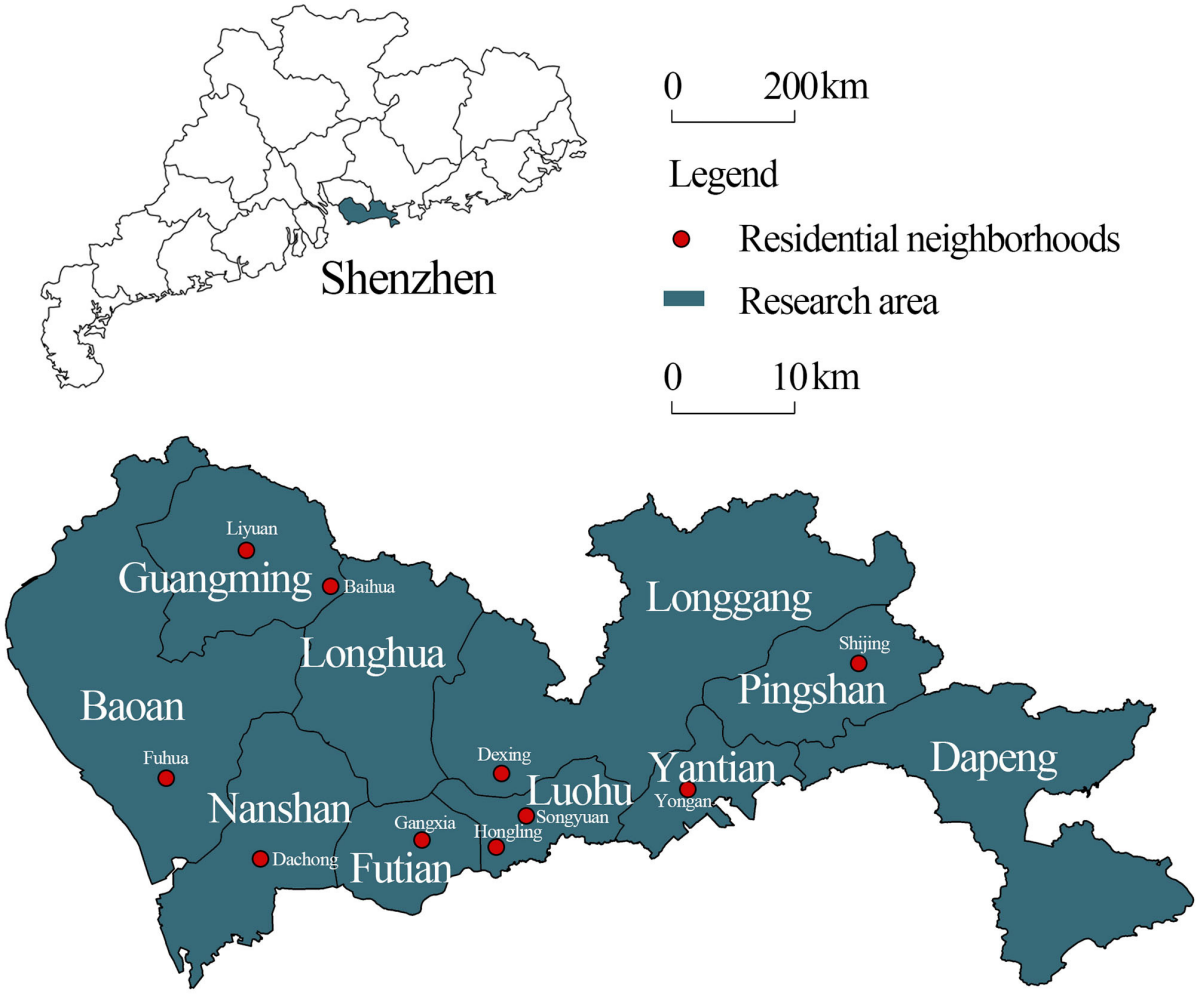
Map of Shenzhen and the locations of the 10 selected neighborhoods.

Data for this study were derived from a questionnaire survey of Shenzhen residents in 2017. A total of 1,000 questionnaires were distributed, and 993 valid copies were returned (response rate: 99.3%). A mix of quantitative and qualitative methods was adopted. Survey respondents were recruited via a multi-stage, stratified proportionate probability sampling process. In the first stage, 10 residential neighborhoods (*shequ*) were randomly chosen from nine districts (i.e., Nanshan, Futian, Luohu, Yantian, Longgang, Baoan, Guangming, Longhua, and Pingshan), using stratified sampling in which the selection probability was proportional to the population size (Figure 1). In the second stage, we randomly selected 100 households from sampled neighborhoods (i.e., Hongling *shequ*, Songyuan *shequ*, Gangxia *shequ*, Dachong *shequ*, Fuhua *shequ*, Dexing *shequ*, Yongan *shequ*, Baihua *shequ*, Liyuan *shequ*, and Shijing *shequ*) and used a Kish grid to select the member to be interviewed from each household.

The survey solicited respondents' sociodemographic characteristics (i.e., age, gender, education, marital status, employment, Hukou status, and family income); see Table 1. Respondents were 40 years old on average, and 94.96% were married. The sample contained slightly more men (51.86%) than women (48.14%). Regarding education level, less than one-third of respondents (28.50%) had a primary school education, 34.44% possessed a secondary school education, and 37.06% held an undergraduate degree or above. Most respondents were employed. More respondents held a household registration in Shenzhen than not. In terms of annual family income, 4.87% of respondents earned less than 25,000 CNY, most (58.61%) earned between 25,000 and 100,000 CNY, 21.65% earned 100,000–200,000 CNY, and 12.74% earned more than 200,000 CNY.

**Table 1:** Social demographic characteristics of population of this study and Shenzhen.

**Variables**	**Proportion (%)/Mean (Standard deviation)**	
	**Population of this study**	**Total population of Shenzhen (2010)**
**Size**	993	10,358,381
**Age**	40.11	29.89
**Gender**		
Male	51.86%	54.19%
Female	48.14%	45.81%
**Education**		
Primary school or below	28.50%	10.09%
High school	34.44%	71.38%
College/University/Above	37.06%	18.53%
**Marital status**		
Married	94.96%	59.55%
Single/Divorce/Widowed	5.04%	40.45%
**Employment**		
Employed	74.62%	73.21%
Unemployed/Retired	23.18%	-
Students	2.20%	-
**Hukou status**		
Shenzhen Hukou	43.50%	17.80%
No-Shenzhen Hukou	56.50%	82.20%
**Family income (Yuan/Year)**		
Below 25,000	4.87%	-
25,000–100,000	58.61%	-
100,000–200,000	21.65%	-
200,000 and above	12.74%	-
Refused	2.13%	-

### Framework

First, we adopted “Quick Bird-2” high-resolution remote sensing image data to accurately depict the spatial distribution of urban green space in Shenzhen; this program was used to calculate the ratio of green space in each target neighborhood. We employed ArcGIS software to establish a current interpretation map of Shenzhen's green space. Second, we used stratified sampling to survey 993 residents on their mental health and satisfaction with the local green space environment. We measured mental health using the 12-item General Health Questionnaire (GHQ-12) (42), whose items pertain to respondents' psychological feelings over the past week at the time of the survey. Third, we considered the mediating role of residents' satisfaction in the local green space environment between residents' mental health and the environmental impact of urban green space. Finally, we developed multilevel linear models to explore the internal logic and mechanisms related to green space, residents' mental health, and residents' satisfaction with the green space environment.

### Variables

#### Outcomes

Although the term “mental health” has no uniform definition, common measurement scales include the GHQ-12, Self-rating Depression Scale (43), Medical Outcomes Study and 36-item Short Form Survey (44). As noted, we applied the GHQ-12 in this study; this instrument is one of the most widely used methods to assess mental health (45–50). The scale's 12 items relate to respondents' psychological feelings over the past week at the time of the survey (see Table 2). Six items are scored on a 5-point Likert-type scale indicating frequency (1 = “at no time,” 5 = “all of the time”); sample statements include “Loss of sleep over worry,” “Capable of making decisions,” and “Could not overcome difficulties.” The remaining six items are reverse scored on the same 5-point Likert-type scale (1 = “all of the time,” 5 = “at no time”; e.g., “Able to concentrate,” “Playing a useful part”).

**Table 2:** Measured items of constructed dimensions.

**Constructed dimensions**	**Variables name**	**Items**
**Satisfaction with green-space environment** Standardized Cronbach's α= 0.823	RS01	Level of satisfactory with Recreation Facilities
	RS02	Level of satisfactory with Neighborhood Green infrastructure
	RS03	Level of satisfactory with Neighborhood living environment
**Mental health** Standardized Cronbach's α= 0.828	MH01	Able to Concentrate
	MH02	Loss of sleep over worry
	MH03	Playing a useful part
	MH04	Capable of making decisions
	MH05	Felt constantly under strain
	MH06	Couldn't overcome difficulties
	MH07	Able to enjoy day-to-day activities
	MH08	Able to face problems
	MH09	Feeling unhappy and depressed
	MH10	Losing confidence
	MH11	Thinking of self as worthless
	MH12	Feeling reasonably happy

#### Green Space

Scholars have generally used investigator observation records to record green space. However, such records can suffer from severe subjective bias. Therefore, we referred to “Quick bird-2” high-resolution remote sensing image data, with a spatial resolution of up to 0.5 m, to calculate the ratio of green space in each case neighborhood. According to the image characteristics of green space (e.g., color, texture, shape, location, and other properties), we determined the interpretation marks and then analyzed remote sensing images together with the actual circumstances in Shenzhen to obtain vector patch data for urban green space interpretation. We next used ArcGIS to construct a current interpretation map of the city's green space using graphics and corresponding data. We also developed an associated greening information database.

#### Mediators

Residents' satisfaction with Shenzhen's green space environment was based on three types of amenities (i.e., recreational facilities, green infrastructure, and living environment, please refer to Table 2). Satisfaction scores ranged from 1 (“very unsatisfactory”) to 5 (“very satisfactory”). We then performed principal component analysis to extract components for all items and calculated respondents' satisfaction index.

#### Control Variables

Multiple individual- and neighborhood-level variables were taken as control variables. Individual-level covariates were as follows (see Table 3 for details): respondent's age (in years), gender (male or female), educational attainment (e.g., primary school or below), Chinese Communist Party membership (yes or no), marital status (single/divorce/widowed vs. married), Hukou status, annual family income, residence length, housing tenure, and social contact size. The five neighborhood-level control variables in this study consisted of point-of-interest (POI) density, bus station density, population density, social trust, and neighborly interactions. Among them, POI density, bus station density, and population density constituted built environmental indicators, while social trust and neighborly interactions were social environmental indicators.

**Table 3:** The mental health of Shenzhen residents.

**Social-demographic**	**Variables**	**Mental health**		
		**SD**	***T*-value**	***P*-value**
Gender	Male	1.957	−1.352	0.177
	Female	1.959		
Age	Before the 1985s	1.924	1.181	0.238
	After the 1985s	1.967		
Marital status	Married	1.943	−0.919	0.359
	Single/Divorce/widowed	2.025		
Education	No high education	1.908	−6.987	0.000
	College/university/above	1.922		
Hukou status	Shenzhen Hukou	1.940	−0.513	0.608
	Non-Shenzhen Hukou	1.974		
Political status	Party member	1.911	−5.574	0.000
	Non-Party member	1.932		
Entire sample		1.959		

#### Data Analysis

We used multilevel linear modeling to unveil the internal logic and mechanisms among green space, residents' mental health, and their satisfaction with the green space environment. The general form of the multilevel regression model can be expressed as follows:

$$\begin{array}{l} Y_{ij}=\beta_{0}+\alpha X_{ij}+\beta Z_{j}+\mu_{j}+\varepsilon_{i} \end{array}$$

Where *Y*_*ij*_ is the mental health index of resident *i* of neighborhood *j*, *X*_*ij*_ represents individual-level variables of resident *i* of neighborhood *j*, and *Z*_*j*_ denotes neighborhood-level variables. α and β denote variation coefficients of individual-level variables and neighborhood-level variables, respectively. μ_*j*_ is the random effect of unobservable factors at the neighborhood level, ε_*i*_ represents the random effect of unobservable factors at the individual level, and β_0_ is a constant.

Variant between-group differences (i.e., between neighborhoods) should be considered during model selection; a multilevel regression model should be adopted if groups exhibit large differences. Therefore, we introduced the dependent variable (i.e., no independent variable was entered) and calculated the intra-class correlation coefficient (ICC) as follows:

$$\begin{array}{l} ICC=\frac{\sigma_{b}^{2}}{\sigma_{w}^{2}+\sigma_{b}^{2}} \end{array}$$

Where $\sigma_{b}^{2}$ represents the variance between neighborhoods, and $\sigma_{w}^{2}$ denotes individual variance within the neighborhood. A larger ICC value indicates that the inter-group variance is significantly greater than the intra-group variance, demonstrating that residents' mental health in the same neighborhood is correlated. In this case, a multilevel model is justified. On the contrary, if the inter-group variance is small and the ICC value is small or close to zero, single-level regression would be more appropriate. Between-group differences could not be ignored in our study; therefore, we chose a multilevel regression model.

## Results

### Relationship Between Green Space and Mental Health

We conducted multilevel linear regression modeling to quantify the effects of individual attributes and the residential environment. Variance inflation factors (1.99) were lower than 3, and the ICC for the null model (0.122) suggested that the residential environment accounted for 12.2% of the variance in residents' mental health. Table 4 summarizes the correlation between green space and mental health. Statistically significant and positive associations between the ratio of green space and residents' mental health implied that the higher the ratio, the better residents' mental health. This finding is consistent with our initial expectations and common sense. First, according to restoration theory, the inherent quality of green space can enhance health or well-being; simply viewing green space can elicit positive effects (51). Second, green space is associated with a healthier environment, including lower air pollution and noise (52, 53). Third, green space can promote mental health by providing an area for sports activities and social interaction, as indicated in prior studies (54, 55). Among neighborhood-level covariates in this study, a negative correlation emerged between population density and mental health. Conversely, POI density, bus station density, social trust, and neighborly interactions each positively influenced mental health with social trust being most noteworthy: social trust can foster residents' mental health, although this correlation is difficult to verify through casual observation. In terms of individual-level variables, gender, higher education, party membership, income, home ownership, and social contact size all had significant positive effects on Shenzhen residents' mental health.

**Table 4:** The association between green space and mental health.

	**Mental health**			
	**coef**.	**S.E**.	***T*-value**	***P*-value**
**Neighborhood-level variables**				
Green space rate	0.226	0.114	(1.99)	0.047^**^
POI density	0.120	0.152	(0.79)	0.432
Bus station density	0.103	0.153	(0.69)	0.488
Population density	−0.060	0.108	(−0.56)	0.578
Social trust	0.208	0.067	(3.12)	0.002^***^
Neighborly interactions	0.065	0.196	(0.33)	0.740
**Individual-level variables**				
Age (reference group: <35 age cohort)				
35 ~ 45 age cohort	−0.092	0.131	(−0.70)	0.482
>45 age cohort	−0.091	0.209	(−0.44)	0.662
Gender (reference group: male)	0.192	0.112	(1.71)	0.088^*^
Education (reference group: lower education)				
Higher education	0.388	0.202	(1.92)	0.055^*^
Middle education	0.086	0.140	(0.61)	0.539
Party membership (reference group: non-party membership)				
Party membership	0.544	0.195	(2.79)	0.005^***^
Marital status (reference group: married)				
Single/Divorce/Widowed	0.154	0.280	(0.55)	0.582
Hukou status (reference group: non-Shenzhen Hukou)				
Shenzhen Hukou	−0.056	0.145	(−0.39)	0.698
Income	1.223	0.469	(2.61)	0.009^***^
Residence length	−0.025	0.092	(−0.27)	0.787
Housing tenure (reference group: renter)				
Homeowner	0.618	0.212	(2.91)	0.004^***^
Social contact size	0.172	0.090	(1.92)	0.054^*^
Constant	−0.560	0.182	(−3.07)	0.002^***^
Observations	993			

* *p <0.1*,

** *p <0.05*,

*** *p <0.01*.

### Relationship Between Green Space and Mediator

The relationship between green space and residents' satisfaction with the local green space environment is outlined in Table 5. Results revealed a significant positive association between these two dimensions. The independent variable (green space) had significant positive effects on predicting residents' satisfaction with the green space environment. Specifically, a 1-point increase in green space produced a 0.438-point increase in residents' satisfaction with the green space environment. This trend suggests that residents living in neighborhoods with relatively more green space are more likely to express greater satisfaction with the green space environment.

**Table 5:** The association between green space and resident's satisfaction with green-space environment.

	**Satisfaction with green-space environment**			
	**coef**.	**S.E**.	***T*-value**	***P*-value**
**Neighborhood-level variables**				
Green space rate	0.438	0.152	(2.89)	0.004^***^
POI density	0.676	0.276	(2.45)	0.014^**^
Bus station density	−0.146	0.172	(−0.85)	0.396
Population density	−0.355	0.275	(−1.29)	0.198
Social trust	−0.093	0.134	(−0.69)	0.490
Neighborly interactions	−0.100	0.220	(−0.46)	0.649
**Individual-level variables**				
Age (reference group: <35 age cohort)				
35 ~ 45 age cohort	−0.182	0.097	(−1.88)	0.060^*^
>45 age cohort	−0.010	0.140	(−0.07)	0.944
Gender (reference group: male)	0.018	0.117	(0.16)	0.877
Education (reference group: lower education)				
Higher education	0.139	0.148	(0.94)	0.350
Middle education	−0.001	0.090	(−0.01)	0.991
Party membership (reference group: non-party membership)				
Party membership	0.297	0.127	(2.35)	0.019^**^
Marital status (reference group: married)				
Single –person	−0.216	0.163	(−1.32)	0.186
Hukou status (reference group: non-Shenzhen Hukou)				
Shenzhen Hukou	−0.100	0.087	(−0.15)	0.252
Income	−0.052	0.135	(−0.39)	0.699
Residence length	−0.014	0.077	(−0.19)	0.581
Housing tenure (reference group: renter)				
Homeowner	−0.062	0.185	(−0.33)	0.739
Social contact size	0.035	0.047	(0.75)	0.455
Constant	0.206	0.163	(1.26)	0.207
Observations	993			

* *p <0.1*,

** *p < 0.05*,

*** *p <0.01*.

With respect to neighborhood-level covariates, POI density had a significant positive impact on residents' satisfaction with the green space environment. Such density reflects the abundance of community green space: the higher the POI density, the greater the community's green space. As mentioned, green space also offers residents an area for leisure and recreation, thus increasing satisfaction with the green space environment. By contrast, bus station density, population density, social trust, and neighborhood interaction adversely affected residents' satisfaction with the green space environment. This pattern may have manifested because some individuals prefer to enjoy green space without being disturbed by others. On an individual level, respondents between 35 and 45 years old demonstrated lower satisfaction with Shenzhen's green space environment, whereas party membership status was significantly positively correlated with residents' satisfaction. These demographic factors may affect people's use of green space, thus shaping their overall satisfaction with the green space environment, as demonstrated in past work (55).

### Mediating Effect of Residents' Satisfaction With Green Space Environment

Residents' satisfaction with the green space environment was associated with their mental health scores, and the correlation between greenness and mental health became non-significant after adding residents' satisfaction into the regression model (see Tables 6, 7). This finding suggests that the relationship between greenness and mental health may be mediated by residents' satisfaction. Compared with Table 4, the impact coefficient of the rate of green space on residents' mental health declined from 0.226 to 0.113. In other words, the mediator (i.e., residents' satisfaction with the green space environment) played a mediating role and indirectly suppressed the direct effect of the independent variable (i.e., ratio of green space) on the dependent variable (i.e., mental health). This trend suggests that residents' satisfaction is likely to influence the relationship between green space availability and mental health. Accordingly, green space indirectly affects mental health.

**Table 6:** Mediation effect for residents' mental health.

	**Mental health**			
	**coef**.	**S.E**.	***T*-value**	***P*-value**
**Neighborhood-level variables**				
Green space rate	0.113	0.107	(1.06)	0.290
POI density	−0.028	0.176	(−0.16)	0.876
Bus station density	0.140	0.128	(1.09)	0.275
Population density	0.006	0.122	(0.05)	0.964
Social trust	0.226	0.065	(3.48)	0.000^***^
Neighborly interactions	0.084	0.157	(0.53)	0.594
**Individual-level variables**				
Age (reference group: <35 age cohort)				
35 ~ 45 age cohort	−0.052	0.128	(−0.40)	0.687
>45 age cohort	−0.102	0.219	(−0.47)	0.641
Gender (reference group: male)	0.185	0.109	(1.69)	0.090^*^
Education (reference group: lower education)				
Higher education	0.339	0.223	(1.52)	0.129
Middle education	0.085	0.144	(0.59)	0.555
Party membership (reference group: non-party membership)				
Party membership	0.457	0.179	(2.55)	0.011^**^
Marital status (reference group: married)				
Single -person	0.212	0.296	(0.72)	0.474
Hukou status (reference group: non-Shenzhen Hukou)				
Shenzhen Hukou	−0.055	0.145	(−0.38)	0.705
Income	1.261	0.468	(2.69)	0.007^***^
Residence length	−0.007	0.084	(−0.08)	0.935
Housing tenure (reference group: renter)				
Homeowner	0.617	0.200	(3.08)	0.002^***^
Social contact size	0.167	0.079	(2.13)	0.033^**^
**Mediator variable**				
Residential satisfaction	0.260^***^	0.051	(5.09)	0.000^***^
Constant	−0.572	0.198	(−2.90)	0.004^***^
Observations	993			

* *p < 0.1*,

** *p < 0.05*,

*** *p < 0.01*.

**Table 7:** Goodness-of-fit of model.

	**Entire sample**
Sobel	0.008^***^
Proportion of total effect that is mediated	1.138
Ratio of indirect to direct effect	0.278

*** *p <0.01*.

Among community-level covariates, social trust was found to have a major effect on mental health. On an individual level, respondents' gender, party membership, income, home ownership, and social contact scale each had significant positive impacts on mental health. Gender is known to influence health-related lifestyles and may be particularly important in green space access (56); notably, gender affects individuals' perceptions and use of the environment. The discrepant impacts of party membership on the environment and mental health may be due to differences in lifestyle and work habits or to the inclusion of groups in certain environments. Income positively affects individuals' mental health because vulnerable communities (e.g., where people earning low incomes reside) lack the economic resources and political power required for environmental risk control. This weak social support can exacerbate residents' fragile mental states; for example, poverty has been shown to be affected by individuals' mental health (57, 58). Additionally, greater social contact has been tied to a stronger sense of trust, belonging, and acceptance, which may promote mental health benefits (38).

## Conclusion and Discussion

China's urban population has ballooned amid swift urbanization. As the percentage of people living in urban environments continues to rise, increasingly dense urban areas have begun to threaten residents' mental health. A better understanding of the various potential benefits of urban green space on residents' mental health is urgently needed and warrants careful consideration. The present study was inspired by a paucity of robust evidence vis-à-vis the link between mental health, residents' satisfaction, and green space. We explored the robustness of these associations and how green space is associated with mental health. Specifically, this study aimed to investigate whether Shenzhen residents' satisfaction with the local green space environment mediated the association between green space and mental health. Questionnaire survey data were gathered from 993 Shenzhen residents in 2017, and “Quick Bird-2” high-resolution remote sensing image data were adopted to calculate the ratio of green space in each case neighborhood and build a green space map of the area. A corresponding information database was then established in ArcGIS. Finally, residents' mental health was evaluated based on the GHQ-12. The novelty of this study is 2-fold. First, we assessed the mediation effect of residents' satisfaction with the green space environment on the relationship between urban greening and mental health in a frontier city in China. Second, we referred to multiple data sources and multi-level data structures to draw conclusions; that is, we considered questionnaire data along with remote sensing data that had a higher spatial resolution compared to the Normalized Difference Vegetation Index used in Liu et al.'s study (4). This multi-level data enabled us to investigate community attributes as well as individual (resident) attributes.

This study offers several valuable insights. First, previous research indicated mixed results regarding the correlation between natural environments and self-rated health (36). Besides Liu et al. (4), our findings provide additional empirical support for the potential mediating role of residents' satisfaction in the relationship between green space and mental health, unveiling that a neighborhood's relative extent of green space can directly improve residents' mental health. These patterns also align with earlier work indicating that neighborhood green space can reduce the risk of mental illness and thus enhance mental health (59–62). Second, our results imply that, different from physical health, individuals' mental health is more implicitly affected by their surrounding environment. The internal mechanism of this influence is complicated, and more research is needed to test and clarify possible mediators (63). Third, the present study bridges a knowledge gap in research on the association between the relative extent of green space, residents' mental health, and potential mediating mechanisms between them in a Chinese city other than Beijing (36) or Guangzhou (4). Because data related to green space and health were derived from different sources in this study, our sample was less susceptible to single-source bias. We also used objective measures of green space based on remote sensing data to promote the reliability of our findings. New technological tools such as “Quick Bird-2” high-resolution data, together with responses to the GHQ-12, produced diverse information to offer an objective, standardized approach for validation studies, as mentioned in prior research (51). Fourth, our results highlight the importance of considering subjective assessments of green space quality (e.g., from residents' points of view) with respect to health benefits. These findings offer potential guidance for stakeholders in pondering environmental justice relative to the provision of green space. Fifth, the relationship between green space, mental health, and residents' perceived satisfaction may vary across cultures or regions. Our mediation analyses showed that, against the backdrop of China's rapid urbanization (e.g., in Shenzhen), residents' satisfaction with the green space environment played a mediating role and indirectly suppressed the direct effect of green space on mental health. These findings enrich knowledge of the mechanisms between green space and mental health.

The results of this study can inform policies promoting the use of green space. Specifically, governments should provide more and better public service facilities, parks, and green space through community planning and construction. Government personnel should also aim to expand community organizations to encourage neighborhood interaction, thereby improving residents' mental health. In addition, given our finding that the relationship between the relative scope of green space and residents' mental health is mediated by residents' satisfaction with the green space environment rather than by its direct health effects, policymakers should consider not only the spatial distribution and quantity of such space but also residents' actual experiences and subjective satisfaction with green space environments when designing green space. Moreover, municipal governments should prioritize harmonious social interaction by allocating public green resources to improve residents' mental health and subjective well-being. As such, site selection should involve an evaluation of the potential merits of land value as well as the factors affecting residents' mental health and well-being. Decision makers can use this knowledge to improve the effectiveness of their mental health interventions as well. This study underlines the need to address subjective perceptions by revealing that a considerable proportion of the impact of mental health status can be explained by residents' satisfaction with green space exposure.

Despite its revelations, this study has several limitations that leave room for future work. First, a major limitation involved selection bias: the associations we have reported could be partially attributable to the selected city (i.e., Shenzhen) and the green space within it. Although we sought to minimize this constraint by considering respondents' socioeconomic and demographic characteristics, our estimations may nonetheless be biased. Moreover, urban green space is not equitably distributed in all cities. In Beijing, for example, green spaces are largely segregated by gated communities, and developers have transformed several public parks into community gardens or golf courses that residents cannot access for free. Such environmental disparity caused by spatial heterogeneity could greatly affect residents' satisfaction with green space. Accordingly, satisfaction may vary regionally, leading to variable mental health outcomes. A natural extension of this study would be to replicate our analysis of the relationship between green space and mental health in other geographical regions. Additional analyses, including taking all green space within a certain distance into account (64), can be performed to delineate the roles of residents' emotions on mental health. Second, our study involved a cross-sectional design and thus precluded clear causal inferences related to possible mediators. Compared with panel data, estimation performance based on cross-sectional data is lower. Longitudinal research could more firmly establish causality around the identified buffering effects of mental health. Third, we used “Quick Bird-2” high-resolution remote sensing image data, with a spatial resolution of up to 0.5 m, to calculate the ratio of green space in each case neighborhood. Using data sources with a higher spatial resolution would be intriguing. Fourth, global positioning system devices could be adopted to evaluate participants' time spent visiting green spaces more precisely than self-report measures. Fifth, because our work was exploratory, the research design was relatively simplistic. Other quality-based characteristics of green space (e.g., aesthetics, biodiversity, walkability, sport/play facilities, safety, and organized social events) have been suggested to consider when predicting green space use (65) and may have influenced our analyses of the mediating role of residents' perceived satisfaction. It would be interesting to investigate whether such factors can lead to causal relationships, especially when some of them are controlled for during mediation analysis (66). Sixth, scholars should continue to explore how demographic variables such as personal race, age, and income contribute to green space use and associated mental health benefits (6, 27). We encourage additional investigation into how and why the effects of green space on mental health vary demographically. We also recommend that researchers consider the impacts of environmental interventions on individuals' mental health outcomes. Doing so may offer valuable insight into which types of residents will benefit most from green space to help stakeholders exploit these advantages more fully. Subsequent work should shed more light on the mechanisms behind the association between green space and mental health and to what extent perceived emotions play causal roles in these relationships.

## Data Availability Statement

The original contributions presented in the study are included in the article/supplementary material, further inquiries can be directed to the corresponding author/s.

## Ethics Statement

Ethical approval was not provided for this study on human participants because Ethical approval for this study was not required in accordance with local legislation and national guidelines. The patients/participants provided their written informed consent to participate in this study.

## Author Contributions

TZ made substantial contributions on the research idea, research design, and English proofreading. YQ contributed to this paper on data acquisition, audit of data analysis, discussions, and implications. ZC contributed to this paper in research background, data analysis, manuscript drafting, and reference cross-checking. YC contributed to this paper in data interpretation, manuscript corrections, and response to the reviewers. All authors contributed to the article and approved the submitted version.

## Conflict of Interest

The authors declare that the research was conducted in the absence of any commercial or financial relationships that could be construed as a potential conflict of interest.
